# What Works in Home-Start According to Parents and Volunteers

**DOI:** 10.1007/s10566-025-09855-7

**Published:** 2025-03-06

**Authors:** A. M. C. Lange, M. Zandbergen, A. M. E. Bijlsma, G. J. Overbeek, L. Boendermaker

**Affiliations:** 1 Centre for Applied Research on Social Sciences and Law, Amsterdam University of Applied Sciences, Postbus 15776, Amsterdam 1001 NG, The Netherlands; 2 Department of Child Development and Education, University of Amsterdam, Amsterdam, The Netherlands; 3 Research Institute Child Development and Education, University of Amsterdam, Amsterdam, The Netherlands

**Keywords:** Home-visiting program, Qualitative research, Effective components, Volunteers, Parenting support

## Abstract

**Background:**

Home-visiting programs often aim to improve parenting skills, parent-child relationships, and children’s developmental outcomes for at-risk families. Although research has identified what elements of these interventions are effective when provided by professionals, little is known about effective components of volunteer-based home-visiting programs.

**Objective:**

This study focused on Home-Start, a preventive home-visiting program, delivering informal social support through volunteers to families with children up to 17 years old struggling with common parenting issues. The aim was to develop a detailed understanding of the core components of the Home-Start and thereby develop a better understanding of the unique elements of volunteer-based home-visiting programs.

**Methods:**

We interviewed 10 parents and 11 volunteers and used thematic analysis.

**Results:**

We found evidence for the relevance of the four principles identified by Home-Start the Netherlands. These are: Needs-oriented care, Focusing on empowerment, Equality and trust, and the Gift of time. We also describe a fifth theme, namely Professional support for the volunteer. The findings suggest overlap with effective components for professional-based support, but also highlight unique elements of volunteer-based home-visiting programs, which are rooted in the shared parenting experiences of volunteers and parents.

**Conclusions:**

This paper provides new insights into the unique value of volunteer-based support for families.

**Supplementary Information:**

The online version contains supplementary material available at 10.1007/s10566-025-09855-7.

## Introduction

Parenthood can be stressful and requires regular readjustment to new life situations, certainly during early parenthood (Martins, 2019). A meta-synthesis of qualitative literature on first-time parents showed that such parents would like access to professional support when needed and want to learn from other parents (Entsieh & Hallström, 2016). Home-visiting programs are an effective tool to support families in need of such support, alleviating the need for more intensive intervention later down the line. Home-visiting programs often, but not exclusively, target families with young children aiming to improve parenting skills, parent-child relationships, and children’s developmental outcomes, for example by advising on parenting techniques, providing social support to parents and supporting parents to access other services and resources (Filene et al., 2013; Gubbels et al., 2021; Leijten et al., 2018; Mortensen & Mastergeorge, 2014; Piquero et al., 2016). Home-visiting programs can best be considered a method of delivery rather than a specific intervention with a specific theoretical background. While they share the belief that targeting parents can improve outcomes for children and that accessibility of services is increased by visiting families in their homes, the exact shape and size of programs vary widely, including the intensity and duration of the intervention, the type of families and outcomes that are being targeted and whether the support is provided by professionals or volunteers (Howard & Brooks-Gunn, 2009). We focus on volunteer-based home-visiting programs targeting parenting, using Home-Start as an example.

Home-Start is a home-visiting program working with a local community network of trained volunteers helping families with children through challenging times. The aim is to provide informal social support to parents with children of up to 17 years of age struggling with common parenting issues. Volunteers visit families on a weekly basis for multiple hours for up to a year. Home-Start in the Netherlands has formulated four principles that guide the support provided. These are needs-orientated care (i.e., needs of families are central to the support provided), focusing on empowerment (i.e., families are empowered), equality and trust (i.e., volunteers collaborate with families on a basis of equality and trust), and the ‘gift of time’ (i.e., volunteers are available to the family for several hours a week for a whole year) (Hollander, 2019). Volunteers for Home-Start in the Netherlands receive an initial training, with a focus on these four principles, before starting to support families. They support one family at a time and receive further support from a coordinator through individual- and group supervisory meetings. A study surveying 384 Home-Start volunteers in the Netherlands found that almost all volunteers were women (96%) and that the average age was 52 years. Just over half of the volunteers (57%) did not have a paid job beside their voluntary work (Smit, 2013). Home-Start has been shown to strengthen parental self-efficacy and parental competencies, improve parenting behavior, and reduce child problem behavior (Asscher et al., 2008a, b; van Aar et al., 2015). Moreover, Dekovič and colleagues (2010) showed that Home-Start’s targeting maternal sense of competence led to improved parenting practices.

In recent years, research has increasingly focused on understanding the effective components of such interventions, rather than only evaluating the effectiveness of the interventions themselves. By identifying effective components, we can improve our understanding of why certain interventions are effective. Moreover, this knowledge can be used to improve care-as-usual, as usually interventions provided to families, children and adolescents are not evidence-based (for example, the Dutch database for effective youth interventions has recognized 225 interventions, of which only 8 have the highest level of evidence and 152 are based on theoretical evidence only). For Home-Start, research into effective components has not yet been carried out. More generally, research on components has mostly focused on professional-based interventions. This study will aim to develop a better understanding of the effective components of volunteer-based home-visiting programs such as Home-Start, as effective components may be different for volunteer-based support as compared to professional-based support.

### Effective Components of Home-Visiting Programs

Although the effective components of Home-Start have not yet been evaluated, multiple meta-analytical studies have examined what components of home-visiting programs in general^1^ are effective in improving positive outcomes for families. These studies have identified various components that yield larger effects, namely (1) supporting parents to use effective parenting strategies (e.g., positive reinforcement such as praising, behavior management techniques such as a time-out, and being responsive and sensitive to a child’s needs), (2) support parents to develop realistic parental expectations of parenthood and child development, (3) using video-based feedback and role-playing as a means of teaching skills to parents, and (4) being home-based rather than inviting families to the office (Filene et al., 2013; Gubbels et al., 2021; Kaminski et al., 2008; Leijten et al., 2018).

Of these meta-analytical studies, only one provided information on the type of home-visitors. In this study, the majority of the included studies (> 90%) were provided by professionals and/or paraprofessionals (Gubbels et al., 2021). Although this study showed that the type of home-visitor did not influence the effectiveness of the interventions, it did not evaluate whether the effective components identified were different across types of home-visitors. Thus, currently, there is no evidence on what is effective when volunteers deliver such programs. Yet, there is some evidence to suggest that support from volunteers or peers is different from professional support.

There are three main differences between professional and volunteer/peer support. Firstly, volunteer-based support is characterized by different values than professional support. In volunteer-based support, (1) the relationship or connection between the volunteer and the client is based on shared experiences, (2) volunteers use experiential rather than formal knowledge, and (3) volunteers develop a more reciprocal relationship with their clients (White et al., 2020). Secondly, volunteer-based support may be suited to different situations than professional support. For example, research has suggested that volunteer-based programs may be a valuable source of social support, but may be less appropriate when there are considerable concerns (e.g., safety of children) that should be addressed by professional services (Byrne et al., 2016; Goff et al., 2023; Warner, 2019). Finally, recent evidence from meta-analyses and reviews suggests that peer- or volunteer-based support mainly has a positive impact on parental and family psychosocial outcomes, such as emotional wellbeing, and feelings of competence and empowerment, rather than on psychopathological outcomes, such as psychiatric symptoms (Byrne et al., 2016; White et al., 2020).

Given the different values, appropriate situations and outcomes associated with professional- and volunteer-based support, differences in effective components between these programs are to be expected. This is supported by qualitative evidence from a study of adult peer-mental health support (Gillard et al., 2015). Although the effective components found in this study could be described as similar to those in professional-based support, such as a trusting relationship and engaging clients with other services, the authors showed that these components were subtly but crucially different when provided by volunteers. As described by White and colleagues (2020), an important difference was that volunteer-based support relied on the shared lived experience of both the client and the volunteer. According to clients and providers, shared experience played a supporting role in developing a trusting relationship, provided hope for the future and reduced stigma. This then served as a bridge to support clients to engage with other services and with the community (Gillard et al., 2015).

The above studies have mainly focused on one-to-one adult peer-support. Yet home-visitors often interact with the broader family, for example by playing with the children to model parenting techniques. The single review of components of volunteer-based home-visiting programs we found (Byrne et al., 2016) coded the frequency of common program components in 43 studies on volunteer-based home-visiting programs. This study found that volunteer-based programs usually (1) provide emotional support (e.g., listening), (2) provide practical support (e.g., household chores), (3) support contact with other services, resources or people, (4) use modelling as a technique to teach new skills, and (5) support parents in their parenting (e.g., providing advice on parenting techniques) (Byrne et al., 2016). However, as the study by Gillard and colleagues (2025) showed, differences between professionals and volunteers may not necessarily lie in using different components, but in the way these components are executed. As the study by Byrne and colleagues (2016) was a quantitative review focusing on common program components only, they could not explore this.

### The Current Study

This study aimed to develop a better understanding of the core components of the volunteer home-visiting program Home-Start, through interviews with Home-Start parents and volunteers. As such, we aim to provide a richer understanding of the potentially unique value of volunteer-based support in home-visiting programs.

## Methods

### Participants

Semi-structured interviews were conducted with 10 parents (2 fathers and 8 mothers) and 11 volunteers (2 men and 9 women), that were selected through convenience sampling (see below for more details). The parents were between 32 and 42 years old (M = 40, SD = 1.35). Not all parents provided the requested demographic information. Of the parents that did provide information, one parent was Dutch and five parents had a non-Dutch background. Three parents had a higher educational degree (University or University of Applied Sciences) and five parents had a practically-oriented educational degree or no educational degree. Families had 1–3 children, who were between 2 and 14 years old. Volunteers had supported between 2 and 10 families with Home-Start prior to the interview. As volunteers usually support one family at a time for approximately a year, this meant that volunteers had approximately 2–10 years of experience. Volunteers and parents were recruited across the country and lived in both urban and rural areas.

### Compliance with ethical standards

The data collection in this study was approved by the Ethics Committee of [anonymized]. All participants signed an informed consent form prior to the interview, were asked for permission to record the interview and were informed that all personal data was anonymized for this study. The authors did not have any conflicts of interests.

### Procedure

Participants were recruited by local Home-Start program coordinators in the Netherlands. In 2022 there were 150 program coordinators across 168 communities and supporting almost 3,000 volunteers (Home-Start, 2022). All participants received an information letter explaining the aim of the study, i.e., understanding ‘what works’ in Home-Start. No information about the researcher was provided to the participants and no prior relationship was established with the participants. The researcher interviewing the participants did sometimes share personal information about her interests or experiences as a parent to ease the interactions during the interview.

Program coordinators were briefed through an information letter and a presentation in an online network meeting by the researchers, providing the opportunity to address questions about the study. The program coordinators then recruited volunteers that had supported at least two families prior to the interview to participate in the study. Volunteers were asked to recruit parents that were at the end of their Home-Start trajectory or finished the program less than a year ago. These inclusion criteria were formed after consultation with several Home-Start program coordinators, to ensure a certain level of experience with the program by the participants. Interested volunteers completed an application form and applied for participation either through their local program coordinator or by directly sending the form to the researchers. Interested parents applied by contacting their volunteer and local program coordinator. All parents who applied were included in the study. As there were more applications from volunteers than could be included in the study, volunteers were selected based on their order of application. However, to achieve proper data saturation, it was checked that a heterogeneous group of volunteers was included based on the residential area (i.e., rural or urban areas) and caseloads of volunteers. We do not know how many volunteers and parents refused.

All applying participants were contacted through telephone and, if not reachable, e-mail to make an appointment for the interview. Interviews with parents were conducted in their home or an alternative location of their preference (e.g., at the local Home-Start agency or online). One parent was interviewed online (through Teams). Parents were also offered a translator if necessary, and were offered to invite their volunteer for joining the interview to make them feel more comfortable, but none of the parents made use of those measures. Interviews were conducted in Dutch or English, depending on the preference of the parent.

Volunteers were interviewed in an online Teams meeting, or in-person at their request. One volunteer requested to be interviewed in-person. For two of the participating parents, their matched volunteer participated in the study too. Interviews and analyses were conducted indepently. All other parents and volunteers were not matched to one another. Interviews lasted for about an hour on average. Upon completion of the interview, all participants received a € 20,- gift voucher for their participation. All interviews were audio-recorded and transcribed before analysis.

The study aimed to include approximately 10 parents and 10 volunteers. After initial analyses of the available data, it was concluded that saturation was achieved and no further interviews were required. We confirmed saturation using the analytical method described by Guest and colleagues (2020). These analyses showed that the first four parent interviews and the first four volunteer interviews included all the main themes and subthemes (11 subthemes in total) described in this manuscript; 100% saturation was achieved with less than half of the sample. Additionally, we conducted a more detailed and rigorous analysis of saturation by including all codes or code groups underlying the subthemes, as these can be seen as describing the subthemes in more nuance. This resulted in a total of 82 code groups. After coding 5 parent interviews and 5 volunteer interviews, saturation was achieved, meaning that adding the fifth parent and volunteer interview added less than 5% of new codes.

### The Interview

The interviewer (MZ) conducted semi-structured interviews using a topic list that was based upon effective components derived from (a) the results of previous studies on home visiting programs, some of which specifically on Home-Start (e.g., Sweet & Appelbaum 2004; Gubbels et al., 2021; Casillas et al., 2016; MacLeod & Nelson, 2000; Hermanns et al., 2013; Asscher et al., 2008a), (b) the Home-Start handbook for volunteers, and (c) knowledge and experience retrieved from a consultation group of experienced Home-Start ambassadors. Participants were asked open ended questions, such as “In your experience, what worked for you in the Home-Start program?” Follow-up questions were asked to acquire more in-depth knowledge, such as “How did you see or notice that? What do you think was the reason that this worked well for you?”. Topics included: what elements of support were most important, the structure of the Home-Start trajectory (activities undertaken, duration, frequency of contact), the relationship with the volunteer, and whether the network or informal support system of the parent had been involved. Volunteers were asked about several additional topics, namely the required attitudes and skills as a volunteer, and the support (i.e., training and supervision) they have received from Home-Start.

### Data Analysis

The qualitative data were analyzed by AL using thematic analysis (Clarke et al., 2015) in MaxQda 22.0.1 (VERBI Software, 2022). The first author (AL) familiarized herself with the dataset through quality checking and reading the interview transcripts. The first author then coded the data by applying labels that described the content of transcript extracts. Extracts could relate to several words or multiple sentences. The third author (AB) independently coded two interviews. The codes of both coders were compared and differences were discussed. The first coder (AL) subsequently continued coding interviews and a second round of double coding occurred, where the third author (AB) coded a third interview. Few differences emerged in this second round.

Using a bottom-up approach, similar codes were combined into themes. In an iterative process, the first author (AL) reviewed these themes by reviewing the coded text, the names of the themes, and the ‘fit’ of the themes to the dataset as a whole. During this process, transcript extracts were relabeled and themes renamed, combined, or divided as necessary.

The four Dutch Home-Start principles were chosen as an overarching framework to order the developed themes. As this framework was familiar to Home-Start volunteers, this method was expected to support the translation of the findings into comprehensible clinical implications. Moreover, a good fit was found between the Home-Start principles and the themes that were developed during the analyses. We added a fifth theme that represented the support that volunteers experienced. Although this is not a core element of the content of Home-Start (i.e., of what is being delivered to families), the interviews suggested this to be relevant for the quality of the care. Indeed, research has shown that the support for professionals is critical in delivering high quality care (Bearman, 2021; Bearman et al., 2017). All themes were dominant themes mentioned by most or all participants.

After finalizing the coding, several validity checks were conducted. First, the content of the overarching themes was compared to available Home-Start material (i.e., the Dutch Home-Start manual, Dutch training material of Home-Start, and published Home-Start reports from the UK). Although some elements were more strongly represented in the interviews or the Home-Start material, this did not led to major disagreements. Therefore, the themes and subthemes were not adapted based on this validity check.

A second validity check involved discussion of the findings with four parents and six volunteers that had been interviewed, as well as several senior coordinators of Home-Start in several meetings organized for this purpose. The parents, volunteers and coordinators felt the themes and subthemes were a good representation of their experiences with Home-Start but also suggested slight changes in labelling or meaning making.

### Team and Reflexivity

The authors of this manuscript are all researchers at a University or University of Applied Sciences, holding either a master’s degree (MZ), PhD (AL and AB) or being a Professor (LB and GJO). They are experienced in the field of effective components, home-visiting programs and/or volunteer-based support programs and had previous experience with qualitative research. All authors are female, expect for GJO being male. All authors are parents. MZ conducted the interviews. AL conducted the main analyses. All authors were involved in reading the manuscript and discussing the structure of the subthemes.

## Results

### Needs-Oriented Care

#### Families’ Needs Are Key

Parents appreciated that volunteers tailored the services to their preferences and needs. One parent described how the starting point of her trajectory was based on their needs, rather than on a protocol. The coordinator subsequently matched parents to a volunteer based on stated preferences or the support required by parents and the skills and experience of volunteers. Volunteers were generally very impressed by the skillfulness of the coordinator in making appropriate matches. Both parents and volunteers stressed the importance of a good match. Hence, volunteers and parents would meet in an initial meeting, after which both the volunteer and the parent were invited to state whether they felt comfortable enough with one another to start a trajectory. This whole process was valued by parents, and proved to parents that their needs were central to Home-Start. As one parent explained:

They give you a chance to express yourself, what you like about the person [volunteer] and what you do not like. They’re helping you to find someone that you’re really comfortable with. It’s not about Home-Start, it’s about us [as parents]. (Parent 10)

This invitation to find an appropriate volunteer contributed to a feeling of a genuine needs-oriented approach. This needs-oriented approach continued throughout the trajectory and directed when, where and for how long the volunteer would visit, but also shaped the content of the visits. The support depended on the needs of the families and could range from emotional and social support (such as listening to parents), to parenting support (such as modelling how to play with the children), supporting families to go out and explore their neighborhood, (i.e. by going to a nearby playground or the library), supporting the children (e.g., with their homework), or practical support (such as helping to read letters or find a job). Home-visitations involved activities that were discussed during intake, but were also adapted to present needs of parents. Parents appreciated the program’s flexibility.

The volunteer is very flexible. She adapts to what I think I need. (Parent 7)

You talk about what they [the families] want to talk about. If they want small talk, that is fine. We do small talk. If they have experienced something difficult in the past week, we talk about that. (Vonlunteer 9)

#### Coaching Attitude

Although we initially named this subtheme ‘following attitude’, one of the parents argued that volunteers do not blindly follow parents’ wishes but rather coach parents. Volunteers explained they needed to stay attuned to the needs and preferences of the family, as the family ultimately decided what they wanted Home-Start to do. However, these needs were not always apparent from the start. Several volunteers described how they needed to carefully listen [to parents] to understand their true needs. Other parents had a long list of issues and needs. In those situations, the volunteer would help the family to create an overview and set priorities. This required asking questions, being curious, empathic, and understanding.

Although priorities could be set, volunteers and parents mentioned that Home-Start did not set treatment goals and that the trajectory would follow the pace of the family. One volunteer explained this as follow:

It is ‘go with the flow’. Within that flow, you try to steer a bit towards this or that. But you do not create a plan, then say ‘this is what we are going to do’, and then do so in phases and steps. (Volunteer 1)

Families said they liked that there was no plan and that the volunteer would adapt the support to their needs. Nevertheless, some volunteers struggled with feelings of being of no help to the family by not achieving concrete outcomes or goals.

Volunteers frequently described how ‘sitting on their hands’ (i.e., refraining from their natural urge to solve things for the family or address problems that may not be experienced by the parents themselves) could be difficult. One volunteer provided an example of how she collected second-hand pieces of furniture for a family, feeling sorry for how poorly they lived, but the family rejected those because she believed that second hand objects might contain ghosts (V5). This was a learning process for many volunteers:

I am very focused on problem solving; I always want to solve 100,000 things at the same time. If things do not go quickly enough, I take over everything. So that was a very big learning process for me, and it still is. (Vonlunteer 11)

### Equality and Trust

#### Non-Judgmental and Respectful Attitude

The previous theme of focusing on families’ needs was closely linked to a non-judgmental and respectful attitude. Both families and volunteers described their preferred characteristics as non-judgmental, respectful, open, friendly, patient and listening. Volunteers explained this involved putting their norms and values aside and respecting families’ choices, even if they disapproved of certain behavior, as values and norms differ between people. *“Those are all norms you bring along*, *what you consider messy or homy. So that openness is very important.” (Vonlunteer 1)* This also meant that the volunteer did not impose their opinion on families. Instead, they would allow families to have or form their own opinion. Families described how this helped to build trust and open up about their lives and problems.

The volunteer does not impose anything, she is very accommodating. I love that. It makes that I also want to open up. Instead of a dominant person who knows everything or think they know better. (Parent 7)

#### Building Trust

Building trust was very important for families and volunteers alike. One parent explained how trust was the key to communication. Trust was achieved by visiting the family on a weekly basis, taking time to get to know one another, truly listening to families, and being non-judgmental and respectful. Sharing own experiences, especially failures, could also help in building trust and invite parents to share their own struggles. Volunteers were usually able to build a strong relationship with the families. Many parents described their volunteer to feel as family or a close friend.

#### Being a Critical Friend

Interestingly, this trusting relationship could then be used by volunteers to seemingly counteract the first theme of focusing on families’ needs; volunteers would sometimes try to raise issues the family might not have raised by themselves, or provide friendly critique on parent’s behavior. Families valued this critical voice, one describing it as having *“a critical friend” (Parent 1)*. One parent explained:

She would also give me a reply, because, of course, she did not always agree with me, or with my opinion in that moment. […] She would be very honest about that. (Parent 8)

Yet, this required tact and positive framing: *“she does not say it in a negative way or in a rude way” (Parent 10).* If volunteers perceived a problem that parents did not perceive, volunteers could try to link this problem to issues the parents *did* want to address, explaining that if they wanted to achieve their own goal, they might also want to think about the problem the volunteer had observed.

#### Collaboration Based on Personal Shared Experiences

Volunteers usually were parents themselves or had worked with children as a professional. As such, they had similar experiences to the parents they supported, which formed an important basis for the support. Hence, parents described volunteers to be accessible and more equivalent to them than professionals. Parents and volunteers talked about going on *“a shared journey” (Parent 1)*, where they would jointly address issues at hand.

Some parents also described how the relationship with the volunteer was characterized by a certain reciprocity. Volunteers choose to get involved in Home-Start for a variety of reasons, including to do something meaningful for society, and enjoying to work with people or children. Parents noticed this inner drive, and described volunteers as helping *“from their heart” (Parent 4)*. Other parents observed how the contact with the family was enjoyable for the volunteers themselves, saying: *“Sometimes the time that you [the volunteer] have to spend has ended*, *but the volunteer still wants to play with them [the children]*, *doing hide and seek.” (Parent 3)*. The fact that parents knew that the volunteer was not being paid to help them and chose to do so for a different reason than to earn an income seemed to add to the feeling of reciprocity and equality experienced by parents.

Nevertheless, parents differed in the extent to which they perceived their relationship with the volunteer to be really equal. When discussing the findings with several parents, one parent observed that they did not feel equal to their volunteer, as the volunteer was much older and more experienced. They did perceive the volunteer to be very approachable and were thankful that the volunteer could coach them in their relatively new life as a parent.

Volunteers would use their own experiences in a variety of ways to coach parents. An important technique used by volunteers was modelling how to play and engage with children. Parents said they paid a lot of attention to what the volunteer did, and learned from the examples given to them.

Home-start talks just very kindly and friendly to the children, in a different way. […] And the children that do not listen to dad and mum, who are small, Home-Start talks so kindly to them, that it goes well. […] It made me happy. I learned from them. (Parent 5)

Afterwards, volunteers would sometimes discuss the situation with the parent to explain what they did. Other volunteers explained that they hoped the parent would copy the playing and join in: *“Giving the example*, *playing together. I think it is most fun when the parent then takes over. That always makes me very happy.” (Volunteer 9)*

Volunteers would also use their experiences to educate parents about parenting issues. They would sometimes do this by sharing their own experiences, but more often, their own experiences were simply the knowledge base they would use to support and guide parents. Sharing tips and advice was mentioned by many parents and volunteers. One parent even said they would write down all the suggestions to keep a record (Parent 1).

### Empowering

#### Normalizing

Volunteers normalized behavior or situations and comforted parents in distress, partly through sharing their own experiences. As one parent explained, this helped them to feel it is okay to make mistakes:

To meet somebody that just could be open and honest with me about her own shortcomings. Because it all worked out; her son is an adult now and he has got his own children and he is successful. She also made mistakes. That it’s okay. (Parent 11)

Other parents explained how talking to their volunteer helped them realize the difficult period with their child would pass.

What Home-Start brought me? […] Trust in my daughter, that it will be okay. At a certain point in time I thought she might have a disorder. It is my first child so I do not have a lot of comparison. (Parent 1)

As a result, parents felt more confident about themselves and their parenting, realizing they *“we’re just normal people” (Parent 11)* and that they were not the only one with these experiences.

#### Positive and Strength-Based

Volunteers involved in many activities that were empowering families. They were positive and strength-based, praising families for the things they were doing well and celebrating small successes. A volunteer explained that a positive an strength-based approach was not about trivializing the impact of a negative situation, but about pointing out the small positive things, for example:

A Syrian girl did not talk. Then we were on the bike and she was singing. I said, ‘Do you hear she is singing?’. The mother was so occupied she did not hear, but the next time she said, ‘She really sings!’ (Volunteer 9)

#### Broadening the Horizon

Volunteers also helped to broaden families’ horizon by taking families outside to jointly explore their neighborhood and get acquainted with nearby facilities such as play grounds, the library or parent-child play groups. As one volunteer explained, these activities helped *“to broaden the parents’ world somewhat.” (Volunteer 9).* Many parents said that Home-Start had helped them to go out more often and that this benefitted them, as well as their children.

They also learned many things outside. When they go somewhere, for example, when we went to the museum, they can still talk about the things they saw there and you see that is expanding their brain to see things for themselves. (Parent 3)

Their horizon was also broadened in a more figurative sense, through talking with their volunteer. Participants explained that the volunteer could provide a whole new perspective, help parents see things in a new light or provide alternative ways of doing things. This often referred to parenting situations, but could also relate to their personal situation. As one parent explained:

We evaluated what happened and then she would come up with examples, advice etc. That gave me a better feeling. It provided a direction. I saw you could do things differently, so it gave me more openings. (Parent 6)

Lastly, the horizon was broadened for some parents by supporting them to learn to speak Dutch or to get acquainted with the Dutch way of doing. This could involve helping parents to find their way to integration courses, or providing information on the school system or Dutch norms and values.

### The Gift of time

#### Social and Emotional Support

Parents and volunteers often referred to the social and emotional support provided by the volunteer. Parents valued to have an additional social contact with whom they could talk, and share their thoughts, feelings and concerns. Some parents were quite isolated, the volunteer being their only social contact, especially parents with very young children or whose family lived far away.

I needed a safety line outside, something else, to not get crazy. […] When I was on maternity leave I only had nursing care at home, mainly, like 99%. I do not have any siblings, my mother lives far away. (Parent 7)

As such, volunteers could temporarily be an addition to a parent’s social network.

#### Bringing Lightness and Fun

Parents and volunteers also talked about the volunteer bringing lightness and fun, with one parent saying: *“The volunteer does things very lightly*, *instead of heavy. For me*, *everything was heavy.” (Parent 1)*. Volunteers made jokes with families, laughed, took them out for activities, played with the kids or just had a good time together. Sessions with the volunteers were also described as low-key. As one parent said: *“There is no pressure to be or say anything profound. Just enjoy the walk in the park.” (Parent 11).*

Parents and volunteers also talked about the volunteer showing up every week, out of their own free will, just for the parent. That was experienced as heart-warming by many parents, and could be an act of kindness which parents were not used to. For example, a volunteer talked about a parent that was pregnant and had had tests run showing the baby was healthy. She started crying when the volunteer congratulated here as no one else had done so. Some volunteers mentioned the importance of their home visits lasting a considerable amount of time and that there was no pressure or time limit. As one volunteer explained:

Listening to- and giving them your attention. I think that is the most important, because it is healing, if people just have time to tell their story, and don’t have to do that within 50 minutes. (Volunteer 9)

Lightness was also brought by providing practical support, such as an extra pair of hands to get things done, or just the presence of a volunteer so parents do not need to do everything on their own. As one volunteer explained:

My experience is that people are very happy if you bring some relief and they do not need to do things on their own. […] Sometimes, you need to help people with some practical things. (Volunteer 10)

There was a wide range of practical support activities that volunteers involved in, including helping parents to acquire things they needed such as bikes, clothing or furniture, supporting parents in their home (e.g., doing chores or tidying up), supporting with taking care of the children, and supporting with administrative or financial tasks (e.g., reading or writing letters or applications).

### Professional Support for the Volunteer

#### Personal Support from the Coordinator

The support from the coordinator was positively valued by all volunteers. The coordinators was perceived as approachable and knowledgeable. Volunteers explained they could call their coordinator to discuss difficult situations or ask for advice. They appreciated that their coordinator was a professional and had a different role towards the families than the volunteer had. For example, one volunteer explained how they could stay in their role of being a support to the family while the coordinator could interfere if that was deemed necessary.

The support provided by the coordinator was unstructured and often depended on the volunteer calling the coordinator. Volunteers described this to be personalized to their needs, although one volunteer also described the danger of calling too little, for example because they would feel this was not necessary, whereas they might actually benefit from the available support.

#### Group Supervision as a Mixed Source of Support

Several times a year volunteers take part in group supervision. Volunteers value these meetings in different ways. Some really enjoyed the meetings. They liked sharing experiences with other volunteers and enjoyed the social aspect. For others, however, the meetings did not benefit them in any way as they did not learn anything new. This was mainly mentioned by more experienced volunteers, especially if they were in a team with a high volunteer turnover rate, or with a high representation of younger and/or less experienced volunteers.

## Discussion

The aim of this study was to develop a better understanding of the core components of the volunteer home-visiting program Home-Start, through interviews with participating parents and volunteers. We further tried to discover potentially unique aspects of volunteer-based support. We found evidence for the significance of the Home-Start principles as described by the Dutch Home-Start handbook, namely (1) Needs-oriented Care, (2) Equality and Trust, (3) Empowering and (4) The Gift of Time. Volunteers further described the benefits of the support they receive from the Home-Start organization (5). Below, we will reflect on these components more generally, as well on how these might be tied more broadly to home-visitors being volunteers as opposed to professionals.

### Effective Components in Home-Visiting Programs

The principle of needs-oriented care was perceived by parents and volunteers to be a central component that was relevant throughout the trajectory of Home-Start. This required flexibility and adaptability of volunteers. Providing care that is tailored to the specific needs of clients is increasingly being recognized as an important element of support (Ng & Weisz, 2016). Whereas intervention developers initially focused on adherence and treatment integrity (i.e., delivering a program as intended) to enhance the quality of the intervention delivery, this has shifted to an acknowledgment of flexibility and tailoring as equally important aspects (Mazzuchelli & Sanders, 2010; Owen & Hilsenroth, 2014). Others have called for more rigorous shifts in service delivery, whereby the help is provided from the perspective of the clients regarding their needs, preferences and what would constitute good outcomes (Baart, 2011; Carey, 2016). This may come close to what Home-Start is providing. Support services and interventions may learn from Home-Start on how to accord client-preferences a more prominent place in the treatment process.

The process of matching families with volunteers was also perceived by volunteers and parents as a crucial element and is, to our knowledge, a rather unique feature. Home-Start coordinators put a lot of effort in deciding which volunteer would fit best with a given family. To better understand this process, we sent out a short survey, which was completed by 47 Dutch Home-Start coordinators. The findings showed that matching was most often based on a match between the needs of the family and the skills, knowledge and preferences of the volunteers. 53% of the coordinators also reported using their gut feeling. Preferences for characteristics such as gender or age, practical considerations such as travel distance, or matching on similarities (including cultural background) were mentioned less frequently (< 33%). Most coordinators (89%) said that they could not always find an optimal match. Whereas some coordinators reported that matches initially perceived as suboptimal could turn out very successful, others said they would never go ahead with a suboptimal match as this was bound to fail. To our knowledge, matching does not seem to play a role in professional-based interventions. Moreover, the scarce research on the effectiveness of matching has focused on measurement-based matching (e.g., Boswell et al., 2022) or matching on cultural background (e.g., Chapman & Schoenwald, 2010). Yet, the above-mentioned survey suggests that cultural background is rarely used as a criterion for matching in Home-Start. Moreover, one coordinator explained how a cultural match had turned out disastrously, as rivalry developed based on the rural background of the one, and the urban background of the other. More research is needed to understand the requirements of successful matching and what role this might play in other forms of support.

Surprisingly, strengthening the informal network of families was rarely mentioned by volunteers or parents as a relevant component, apart from sometimes stimulating families to take part in groups or activities to meet new people. This is surprising as the Home-Start training highlights/emphasizes the relevance of this element, as does research. For example, research on interventions for families with multiple problems showed activation of the informal network to be incorporated as important component in six out of eight evidence-based interventions (Visscher et al., 2020b). At the same time, a Dutch study (Visscher et al., 2020a) has found that professionals often struggle to deliver this component. They studied delivery of effective components across various effective interventions for families with multiple problems and showed that activation of the social network was the least-provided element (Visscher et al., 2020a). This has led to an increased emphasis on supporting and teaching professionals various ways of activing the network (see for example, https://awtjf.nl/tools-voor-professionals/reflectie-tools/de-kracht-van-samenwerken/ for a Dutch tool). Research will need to evaluate whether these methods and tools are effective in increasing social network activation in different contexts and whether this is perceived as acceptable and beneficial by families.

### Effective Components of Volunteer-Based Home-Visiting Programs

White (2020) described three unique features of volunteer-based support: a basis in shared experiences, using experiential rather than formal knowledge and a more reciprocal relationship. We found evidence for all three elements. Home-Start volunteers drew on their own experience to support parents in their daily parenting struggles, which is different from professional-based parenting interventions that support parents in acquiring a specified set of evidence-based parenting techniques, using formal knowledge. The shared personal experiences were also described as an important element in supporting the development of trust and supporting a collaborative and reciprocal relationship. Sharing experiences was also helpful in normalizing issues families were experiencing. This latter finding aligns with findings from recent meta-analyses on group peer support programs showing that, through sharing personal experiences, group peer support can lead to improvements in empowerment and self-efficacy, can help to normalize behavior, learn new behavior, and gain new perspectives based on shared experiences (Burke et al., 2019; Moltrecht et al. 2024). Besides the three elements described by White (2020), we feel that the component we labelled ‘Gift of time’ might be specific to volunteers; more specifically the element of bringing lightness and fun. This includedvolunteers ‘giving’ their free time to families. This led to a more reciprocal relationship but also made families feel valued. Furthermore, the time spent on fun activities with the children or the whole family were appreciated and deemed important. This is less commonly provided by professionals due to heavy caseloads and the demands of a professional position (Lushin et al., 2023). Although the gift of time did include family activities, this feature of volunteer-based support does not seem reserved to family peer-support, since giving free time to families and undertaking fun activities together can equally be done in one-to-one peer support. As such, we did not find evidence for aspects that were unique about volunteer-based support to families as compared to one-to-one adult peer-support.

### Clinical Implications

To deliver effective and good quality care professionals and volunteers do not only need to know which elements are effective, but also how these should be delivered; just knowing *what* components should be delivered does not lead to competent and adherent delivery of these components (Blase et al., 2018; Sekreve et al., 2020). Competent and adherent delivery, also called treatment integrity (Perepletchikova, 2011) is related to better outcomes for children and families (Collyer et al., 2019; Goense et al., 2016; McLeod et al., 2013).

The current study provides detailed descriptions of the effective components of Home-Start and thereby provides a basis or a first step for a better understanding of this ‘how-to’. This can be used to support professionals and volunteers in delivering good quality care, for example in supervision. Supervision, especially when including active learning methods and providing formative feedback on the how-to, has been found to improve adherence and client outcomes, and is thus an effective way to improve quality (Johnson, 2019; Schoenwald et al., 2009).

For Home-Start, the authors have developed a card game providing twelve concrete and short descriptions of behaviors or attitudes for each of the four principles. These cards can be used in various ways during supervision to reflect and give feedback on the extent to which volunteers demonstrate these behaviors and attitudes when working with families. Although such targeted supervision on ‘adherent delivery’ is common in some evidence-based professional-based interventions (Albrecht, 2008; Henggeler & Schoenwald, 1999), this is new for volunteer-based support. More research is needed to understand the needs and preferences of volunteers regarding support for them. Conversation with Home-Start coordinators suggests that not all volunteers may want to learn or be open to feedback, as they engage in Home-Start as a volunteer in their spare time. Practices to support volunteers should take this into consideration.

Our research suggests that the one-to-one support provided by the coordinator in a flexible and needs-oriented way is highly valued by volunteers. This seems to be a parallel process to the needs-oriented care provided to families by volunteers (Goense et al., 2015). The experiences with group supervision, however, varied greatly in this study. The interviews suggested that more experienced volunteers benefitted less from group supervision as they felt they did not learn anything new from their participation in supervision. Indeed, the levels of experience may vary greatly between volunteers based on their age, employment background and years of parenting experience. Alternative ways of organizing group supervision could perhaps be developed to address this issue. For example, child protection services in Amsterdam have been trialing a method whereby more experienced supervisees receive a more expert role during the group supervision meetings (Boendermaker & Regeer, 2018). Discussing the application of components during supervision might be an alternative way to address the apparent lack of depth and learning potential described by experienced interviewees in our study. More research is needed to understand the needs of experienced volunteers or professionals and how to optimally support them.

### Limitations and Future Research

This study is based on parents’ and volunteers’ experiences of Home-Start in the Netherlands. Yet, Home-Start is a worldwide organization, operating in 22 countries across the world. We know that programs need cultural adaptations to fit local contexts (Sundell et al., 2016; Olsson et al., 2023) and thus the experiences of parents and volunteers in the Netherlands may differ from those in other countries. The results may also not be representative of all parents and volunteers as selection bias may have led the most enthusiastic parents and volunteers to choose to participate in the study.

We would also like to stress that these findings represent core elements from the perspective of parents and volunteers. Moreover, we focused on elements that parents and volunteers felt were effective. We did not ask about elements that might have been counterproductive. We were further unable to include the perspective of children as they struggled to reflect on the behavior of the volunteer, due to their young age and the fact that volunteers more often supported parents. We also did not evaluate to what extent these components relate to better outcomes. Long-term rigorous research into effective components is needed to shed light on this question. Nevertheless, we would like to underline the value of client perspectives in understanding important components of care. Although recipients of care may not have a full understanding of what components of an intervention or program were responsible for change, it is crucial to understand what matters to clients and ensure we take their perspectives into account.

## Conclusion

This study provided detailed descriptions of the effective components of Home-Start, a volunteer-based home-visiting program. Effective components included needs-oriented care, empowering, trust and equality and the gift of time. We also found evidence for unique aspects of the volunteer-based support, based on shared personal experiences, using experiential knowledge and having a more reciprocal relationship. The time volunteers could spend in fun activities with the children and family may also be a feature that is characteristic for volunteer-based support.

## Electronic supplementary material

Below is the link to the electronic supplementary material.

Supplementary Material 1

## Footnotes

1 We have also included effective components of parenting programs, as one of the major aims of Home-Start is supporting parenting.
