# Supplementary material for: What Works in Home-Start According to Parents and Volunteers
Source: Child Youth Care Forum. 2025 Mar 6;54(5):1157–77. doi: 10.1007/s10566-025-09855-7 (PMC12464112; doi:10.1007/s10566-025-09855-7)
Supplement: Supplementary file 1 — Supplementary Material 1 [file 10566_2025_9855_MOESM1_ESM.docx]

**Appendix 1: COREQ (COnsolidated criteria for REporting Qualitative research) Checklist.**

A checklist of items that should be included in reports of qualitative research. You must report the page number in your manuscript where you consider each of the items listed in this checklist. If you have not included this information, either revise your manuscript accordingly before submitting or note N/A.

| **Topic** | **Item no.** | **Guide questions/description** | **Reported on page no.** | **Tekst in manuscript** |
| --- | --- | --- | --- | --- |
| **Domain 1: research team and reflexivity** | | | |  |
| *Personal characteristics* | | | |  |
| Interviewer/  facilitator | 1 | Which author/s conducted the interview or focus group? | 7 | MZ conducted the interviews. |
| Credentials | 2 | What were the researcher’s credentials? e.g., PhD, MD | 9 | The authors of this manuscript are all researchers at a University or University of Applied Sciences, holding either a master (MZ), PhD (AL and AB) or being a Professor (LB and GJO). |
| Occupation | 3 | What was their occupation at the time of the study? | 9 | See item 2 |
| Gender | 4 | Was the researcher male or female? | 9 | All authors are female, expect for GJO being male. |
| Experience and training | 5 | What experience or training did the researcher have? | 9 | They are experienced in the field of effective components and home-visiting programs and had previous experience with qualitative research. |
| *Relationship with participants* | | | |  |
| Relationship established | 6 | Was a relationship established prior to study commencement? | 6 | no prior relationship was established with the participants. |
| Participant knowledge of the interviewer | 7 | What did the participants know about the researcher? e.g., personal goals, reasons for doing the research | 6 | All participants received an information letter explaining the aim of the study, i.e., understanding ‘what works’ in Home-Start. |
| Interviewer characteristics | 8 | What characteristics were reported about the interviewer/facilitator? e.g., bias, assumptions, reasons and interests in the research topic | 6 | No information about the researcher was provided to the participants. The researcher interviewing the participants did sometimes share personal information about her interests or experience as a parent to ease the interactions during the interview. |
| **Domain 2: study design** | | | |  |
| *Theoretical framework* | | | |  |
| Methodological orientation and theory | 9 | What methodological orientation was stated to underpin the study? e.g., grounded theory, discourse analysis, ethnography, phenomenology, content analysis | 8 | thematic analysis |
| *Participant selection* | | | |  |
| Sampling | 10 | How were participants selected? e.g., purposive, convenience, consecutive, snowball | 6 | Convenience sampling, more specifically:  Program coordinators were briefed through an information letter and a presentation in an online network meeting by the researchers, providing the opportunity to address questions about the study. The program coordinators then recruited volunteers that had supported at least two families prior to the interview to participate in the study. Volunteers were asked to recruit parents that were at the end of their Home-Start trajectory or finished the program less than a year ago. These inclusion criteria were formed after consultation with several Home-Start program coordinators, to ensure a certain level of experience with the program by the participants.  Interested volunteers completed an application form and applied for participation either through their local program coordinator or by directly sending the form to the researchers. Interested parents applied by contacting their volunteer and local program coordinator. All parents who applied were included in the study. As there were more applications from volunteers than could be included in the study, volunteers were selected based on their order of application. However, to achieve proper data saturation, it was checked that a heterogeneous group of volunteers was included based on the residential area (i.e., rural or urban areas) and caseloads of volunteers. We do not know how many volunteers and parents refused. |
| Method of approach | 11 | How were participants approached? e.g., face-to-face, telephone, mail, email | 6 | See item 10 |
| Sample size | 12 | How many participants were in the study? |  | Semi-structured interviews were conducted with 10 parents (2 fathers and 8 mothers) and 11 volunteers (2 men and 9 women) |
| Non-participation | 13 | How many people refused to participate or dropped out? Reasons? | 6 | We do not know how many volunteers and parents refused. |
